# Relationship between tooth loss, low masticatory ability, and nutritional indices in the elderly: a cross-sectional study

**DOI:** 10.1186/s12903-019-0778-5

**Published:** 2019-06-13

**Authors:** Nozomi Okamoto, Nobuko Amano, Tomiyo Nakamura, Motokazu Yanagi

**Affiliations:** 1 Department of School Psychology, Developmental Science and Health Education, Hyogo University of Teacher Education, Simokume 942-1, Kato City, Hyogo Japan; 2 Department of Clinical Nutrition and Dietetics, Konan Women’s University, Morikita-cho 6-2-23, Higashinadaku, Kobe City, Hyogo Japan; 3 Department of Epidemiology, Nara Medical University, 840 Shijo-cho, Kashihara City, Nara Japan; 4 Department of Food Science and Human Nutrition, Ryukoku University, Yokotani1-5, Setaoe-cho, Otsu City, Shiga Japan; 5 Department of Food and Nutrition, Tezukayama University, Gakuenmaeminami 3-1-3, Nara City, Nara Japan

**Keywords:** Tooth loss, Masticatory ability, Serum albumin, BMI

## Abstract

**Background:**

Low masticatory ability and the resulting decrease in intake of masticable foods can result in undernutrition. The present study investigated the relationship between tooth loss, low masticatory ability, and nutritional indices in the elderly.

**Methods:**

The data analyzed in this study were retrieved from the baseline data of the 2007 Fujiwara-kyo study, a prospective cohort study of community-dwelling elderly individuals. Subjects included 1591 men and 1543 women, both with a median age of 71 years. The maximum occlusal force was measured as an objective index of masticatory ability. Foods were divided into five groups based on hardness: Group 1 (bananas, etc.), 0.53 kg; Group 2 (boiled rice, etc.), 1.22 kg; Group 3 (raisins, etc.), 2.93 kg; Group 4 (raw carrots, etc.), 4.38 kg; and Group 5 (beef jerky), 6.56 kg. To obtain a subjective index of masticatory ability, a questionnaire-based survey was conducted to determine whether subjects could masticate foods within each group. As nutritional indices, serum albumin levels and body mass index (BMI) data were used.

**Results:**

The median number of teeth was 21. The proportion of subjects for whom all five food groups were masticable showed a significant decrease in the number of teeth in both males and females. Logistic regression analysis showed that, after adjustment for confounders, no significant relationships were observed between the number of teeth and the masticatory ability with nutritional indices in males. In females, a maximum occlusal force of 100 to 300 N (OR = 1.65; 95% CI = 1.06–2.55) or less than 100 N (OR = 1.95; 95% CI = 1.15–3.31) showed a significant correlation with serum albumin levels below 4.4 g/dL (reference: 500 N or more). In addition, the masticability of all five food groups showed a significant correlation with BMI < 21.0 kg/m^2^ (OR = 0.62; 95% CI = 0.46–0.85) in females.

**Conclusions:**

A low number of teeth was associated with low masticatory ability in both males and females. Low masticatory ability was associated with low plasma albumin levels and low BMI in females. Not smoking, maintaining grip strength, preventing cancer, and masticatory ability are important for preventing undernutrition.

## Background

In elderly people, tooth loss is associated with an impairment of daily activities and an increased incidence of severe diseases. Among people older than 65 years, the incidence of functional disability [1, 2] and mortality rate [3] are higher in those with fewer than 20 teeth. In a 10-year, prospective, cohort study of community-dwelling people older than 60 years, those who were entirely edentulous were more likely to demonstrate reduced walking speeds and cognitive function [4]. The all-cause mortality rate [5, 6], cardiovascular disease-related mortality rate [5, 6], stroke-related mortality rate [6], and stroke incidence [5] were greater in edentulous subjects.

One hypothesis for the connection between the number of teeth and serious disease points to inflammation caused by periodontal disease. Multiple tooth loss is often due to periodontal disease, and the causative bacteria of periodontal disease and inflammatory cytokines formed in periodontal disease have negative effects on blood vessels in the brain and heart. It has been reported that the prevalence of carotid arterial plaque is significantly higher in people who have lost 10 or more teeth [7]. A second hypothesis is that people with low masticatory ability have an insufficient nutrient intake. In this context, the levels of plasma vitamin C, which has antioxidant activities, are lower in edentulous people [8]. In elderly people with multiple tooth loss, it is not unreasonable to expect that low masticatory ability may lead to undernutrition; however, few studies verifying this sequential relationship have been published.

The present research is a cross-sectional study of community-dwelling, independent, elderly people, in which the relationships between tooth loss, low masticatory ability, and nutritional indices were ascertained. For an objective evaluation of masticatory ability [9], the occlusal force was measured. For subjective evaluation of masticatory ability [9], a questionnaire was used to survey whether people could masticate various typical foods. The serum albumin level and BMI were used as nutritional indices.

The following hypotheses were tested in this study:

(i) A smaller number of teeth is associated with lower maximum occlusal force, fewer masticable foods, lower serum albumin levels, and a lower BMI.

(ii) Among people with approximately the same numbers of teeth, people without occlusal support in the functional molar region have a lower masticatory ability, serum albumin levels, and BMI than people with such support.

## Methods

### Subjects

The data analyzed in this study were retrieved from the baseline data of the 2007 Fujiwara-kyo study. The Fujiwara-kyo study is a prospective cohort study, a study of successful aging in the elderly [10–13]. The participants were both male and female volunteers from the Nara prefecture aged 65 years or older at the baseline survey, who were living in their own homes and were able to walk independently. The baseline health examination included a dental examination, measurement of height, body weight, and physical strength, blood collection, and a questionnaire about lifestyle. The dental examination was carried out on 3510 subjects. The current study used only those subjects for whom data on the maximum occlusal force was available. Thus, the current study consisted of 1591 males and 1543 females. Sixty-eight subjects were randomly selected from a total of 3134 subjects to test-retest the reliability of the maximum occlusal force analysis.

### Dental examination

The dental examination methods have previously been reported [14, 15]. The examination was carried out by two calibrated dentists, under artificial light, in accordance with the World Health Organization’s oral examination method (version 4) [16]. The parameters assessed were the number of teeth and presence or absence of prostheses and occlusal support. Remaining teeth were defined as those with roots embedded in the jaw bone, consisting of healthy teeth, caries-affected teeth, and teeth with crowns, inlays, and bridges. Fully erupted third molars were included among the remaining teeth, but remaining roots and highly mobile teeth were excluded. Functional teeth were defined as remaining teeth, pontics, removable dentures, artificial teeth, and dental implants. Dentures that subjects had but did not wear while eating were not considered functional teeth.

Occlusal support for functional teeth was evaluated according to the Eichner classification [17], according to which premolars and molars are counted as one region, with a total of four supporting zones. Individuals classified at rank A have four occlusal contacts in the posterior region, whereas those at rank B/C have zero to three occlusal contacts in the posterior region.

### Measurement of maximum occlusal force by the dental Prescale system

The Dental Prescale System [18, 19] operates as follows:

(i) Depending upon the occlusal pressure, microcapsules in the color-forming layer of a horseshoe-shaped, pressure-sensitive sheet (Dental Prescale 50H Type R; Fuji Film Co., Tokyo) are ruptured, resulting in red coloration as a result of chemical reactions.

(ii) When the Dental Prescale is inserted in a specialized evaluation device, the Dental Occlusion Pressure graph FPD-707 (Fuji Film Co.), the coloration is scanned, the intensity is determined, and the occlusal contact area (mm^2^) and occlusal force (N) are calculated.

Because this was a field examination, not carried out in a clinic, the subjects sat on commercially available tubular folding chairs. The chair’s seating surface is square and 40 cm high. The subjects were instructed to sit with a straight back, legs open to the same width as the shoulders, both hands resting on the front of the thighs, and the lower legs meeting the floor at 90° angles. In order to measure the maximum occlusal force at the intercuspal position, the subject’s head was gently supported so that Camper’s plane, that is, a plane passing through the subnasal point and the two tragia, was horizontal. After a mandibular tapping exercise for 3 s, the Dental Prescale was inserted in the subject’s mouth, its positional relationship with the upper and lower jaws was confirmed, and the subject was instructed to bite as hard as possible for 3 s. For subjects who wear dentures when eating, the measurements were done with the dentures fitted, whereas for subjects who do not wear dentures, the measurements were done without them.

### Questions about masticable foods

The hardness of foods was measured by applying weights using a V-shaped plunger and expressed as the weight needed to shear them. The foods were divided into the following five groups on this basis of hardness [20, 21]:

Group 1: Bananas, corned beef, wafers, tofu; applied weight: 0.53 kg.

Group 2: Boiled rice, boiled asparagus, apples; applied weight: 1.22 kg.

Group 3: Raisins, pickles; applied weight: 2.93 kg.

Group 4: Raw carrots, celery, boiled ham; applied weight: 4.38 kg.

Group 5: Beef jerky; applied weight: 6.56 kg.

Using a questionnaire, subjects were asked whether they were able to masticate all foods included in each hardness group.

### Nutritional indices

Blood was collected from an antecubital vein after an overnight fast. Albumin, triglycerides, low-density lipoprotein cholesterol, high-density lipoprotein cholesterol, fasting plasma glucose, hemoglobin A1c, and creatinine were measured.

The subjects were given hospital gowns of similar thickness to wear, after which height and body weight were calculated using an internal fat meter (Tanita Co., Tokyo), and BMI was calculated.

### Other potential confounding factors

The subjects were asked about their length of education, drinking frequency (hardly drink or drink on at least 1 day a week), and smoking status (never, ex-smoker, or current smoker), using a self-administered questionnaire. Grip strength, which has been reported to have a significant correlation with mortality rate was measured [22]; with the subject seated and with his/her arms hanging naturally, he/she was instructed to grasp a digital grip-strength meter (Takei Co., Niigata) with his/her dominant hand. After approximately 1 min, the measurement was repeated, and the better of the two measurements was used in the analysis.

After sitting quietly for more than 5 min, blood pressure was measured twice at an interval of 30 s, using an automatic blood pressure manometer (ES-P2100; Terumo Co., Tokyo, Japan). The mean of the two measurements was used in the analysis. The medical history judgment method has been reported previously [23]. Cancer, myocardial infarction, and cerebrovascular disease were evaluated based on medical history and current medication. Hypertension was defined based on the Japanese Society of Hypertension criteria [24], that is, by medical history, current use of antihypertensive medications, and/or systolic/diastolic blood pressure at 140/90 mmHg or higher. Dyslipidemia was defined based on medical history, current lipid-lowering medications, and/or one or more of the following blood biochemistry test results in the Japan Atherosclerosis Society guidelines [25]: triglycerides ≥150 mg/dL, low-density lipoprotein cholesterol ≥140 mg/dL, and high-density lipoprotein cholesterol at < 40 mg/dL. Diabetes mellitus was defined based on medical history, current antidiabetic medication, and/or one or both of the following blood biochemistry test results, according to the guidelines of the Japan Diabetes Society [26]: fasting plasma glucose level ≥ 126 mg/dL and hemoglobin A1c level (National Glycohemoglobin Standardization Program values) ≥ 6.5%. For the evaluation of renal function, the estimated glomerular filtration rate (eGFR) was calculated from the serum creatinine, and a value of < 60 was judged as poor [27].

### Analysis methods

As to test-retest reliability of occlusal contact area and maximum occlusal force, measurements were repeated after 5 min, and Spearman’s rank-correlation coefficient and the intra-class correlation were calculated. The Mann-Whitney test and the Kruskal-Wallis test were used for comparison of continuous variables between groups, and the χ^2^ test was used for comparison of categorical variables between groups. For trend tests, the Jonckheere-Terpstra test was used for continuous variables, and the Mantel-extension test was used for categorical variables. Logistic regression analysis by the forced-entry method was carried out with serum albumin levels below the lower quartile and a BMI below the lower quartile as dependent variables, and age, education length, alcohol consumption, smoking status, grip strength, and disease history as the explanatory variables. Goodness of fit was assessed using the Hosmer-Lemeshow test. Statistical analysis was performed using SPSS (version 25; SPSS Japan Inc., Tokyo, Japan). The significance level was set at 5%.

## Results

The medians (interquartile ranges) of the occlusal contact area and maximum occlusal force in the 68 subjects with whom the test-retest reliability of maximum occlusal force was examined were 7.9 (9.9) mm^2^ and 284.1 (354.6) N, respectively, in the first test and 8.9 (11.1) mm^2^ and 296.6 (409.5) N, respectively, in the second. The Spearman’s rank-correlation coefficients between the first and second tests in these 68 subjects were 0.927 and 0.952 for occlusal contact area and maximum occlusal force, respectively, and the intra-class correlations were 0.952 and 0.951 for occlusal contact area and maximum occlusal force, respectively.

Table 1 shows the results for each of the questionnaire items. In all subjects, the lower quartiles for serum albumin levels and BMI were 4.4 g/dL and 20.9 kg/m^2^, respectively. The medians (interquartile ranges) for age, number of teeth, and maximum occlusal force were 71.0 (7.0), 21.0 (17.0), and 302.7 (407.7) N, respectively. In terms of Eichner classification for functional teeth, 87.2% of subjects belonged to rank A. The proportion for whom foods in all five groups were masticable was 79.3%. A comparison between the sexes showed that the maximum occlusal force was significantly greater in males than in females, but no difference in the number of teeth and the proportion for whom group-5 foods were masticable was found between males and females.

**Table 1:** Basic attributes of the study subjects

	All subjects (*n* = 3134)	Males (*n* = 1591)	Females (*n* = 1543)	*p*-value^*^
Age, y	71.0 (68.0, 75.0)	71.0 (68.0, 76.0)	71.0 (68.0, 75.0)	0.308
Education ≤ junior high school, %	883 (28.4)	410 (26.0)	473 (30.9)	0.002
Serum albumin, g/dL	4.6 (4.4, 4.8)	4.6 (4.4, 4.7)	4.6 (4.4, 4.8)	0.003
BMI, kg/m^2^	22.9 (20.9, 24.8)	23.0 (21.1, 24.8)	22.6 (20.8, 24.7)	0.033
Number of teeth	21.0 (9.0, 26.0)	21.0 (9.0, 26.0)	21.0 (9.0, 26.0)	0.341
Eichner class of functional teeth, %				
A	2734 (87.2)	1389 (87.3)	1345 (87.2)	0.455
B	390 (12.4)	195 (12.3)	195 (12.6)	
C	10 (0.3)	7 (0.4)	3 (0.2)	
Occlusal contact area, mm^2^	9.1 (4.3, 16.9)	10.5 (5.1, 18.9)	8.1 (3.8, 14.8)	< 0.001
Maximum occlusal force, N	302.7 (135.1, 542.8)	345.3 (160.1, 604.7)	262.3 (119.3, 479.6)	< 0.001
Foods reported to be masticable, %				
Group 1 (0.53 kg needed to shear)	3113 (99.3)	1581 (99.4)	1532 (99.3)	0.831
Group 2 (1.22 kg needed to shear)	3122 (99.6)	1584 (99.6)	1538 (99.7)	0.528
Group 3 (2.93 kg needed to shear)	2925 (93.3)	1485 (93.3)	1440 (93.3)	0.703
Group 4 (4.38 kg needed to shear)	2837 (90.5)	1442 (90.6)	1395 (90.4)	0.488
Group 5 (6.56 needed to shear)	2529(80.7)	1287 (80.9)	1242 (80.5)	0.808
All five food groups were masticable, %	2484 (79.3)	1269 (79.8)	1215 (78.7)	0.279
Alcohol consumption at least once per week, %	1232 (39.3)	1000 (62.9)	232 (15.0)	< 0.001
Smoking status				
Current smoker, %	326 (10.4)	288 (18.1)	38 (2.5)	< 0.001
Ex-smoker, %	1020 (32.5)	959 (60.3)	61 (4.0)	
Never smoked, %	1788 (57.1)	344 (21.6)	1444 (93.6)	
Grip strength, kg	28.1 (22.7, 35.8)	35.7 (31.4, 39.7)	22.8 (20.1, 25.5)	< 0.001
Hypertension, %	2222 (70.9)	1170 (73.5)	1052 (68.2)	< 0.001
Dyslipidemia, %	1867 (59.6)	888 (55.8)	979 (63.4)	< 0.001
Diabetes, %	495 (15.8)	320 (20.1)	175 (11.3)	< 0.001
Cancer, %	293 (9.3)	186 (11.7)	107 (6.9)	< 0.001
Myocardial infarction, %	80 (2.6)	57 (3.6)	23 (1.5)	< 0.001
Cerebral infarction, %	183 (5.8)	108 (6.8)	75 (4.9)	0.022
eGFR< 60, %	779 (24.9)	443 (27.8)	336 (21.8)	< 0.001

Numerals indicate the median (upper quartile, lower quartile), or number of subjects (%)

*BMI* Body mass index, *eGFR* estimated glomerular filtration rate

^*^The tests for differences between males and females are the Mann-Whitney test for continuous variables, and the χ^2^ test for categorical variables

Table 2 shows the relationships between the number of teeth, masticatory ability, and nutritional indices. In males, the median (interquartile range) maximum occlusal force decreased significantly with the number of teeth (*p* for trend < 0.001), being 546.1 (398.9) N, 248.1 (219.7) N, and 115.0 (145.2) N in subjects with 20 or more, 10 to 19, and fewer than 10 teeth, respectively. The proportions for whom all five food groups were masticable also decreased significantly (*p* for trend < 0.001), being 94.5, 66.0, and 57.4%. There were significant increases (*p* for trend < 0.001) in the proportion of subjects with serum albumin levels below the lower quartile of 4.4 g/dL and those with a BMI below the lower quartile of 21.0 kg/m^2^, with a decreasing number of teeth. In females, the maximum occlusal force and the proportion of subjects for whom all five food groups were masticable decreased significantly with the number of teeth (*p* for trend < 0.001). In contrast with males, the proportion of subjects with serum albumin levels below 4.4 g/dL and with BMI below 21.0 kg/m^2^ showed no significant correlations with the number of teeth.

**Table 2:** Relationships between tooth number, masticatory ability, and nutritional indices

		Number of teeth		Occlusal force, N		All five food groups were masticable		Serum albumin < 4.4 g/dl		BMI < 21.0 kg/m^2^	
	Number	Median (25, 75%)	*p-*value^*^	Median (25, 75%)	*p-*value^*^	%	*p-*value^§^	%	*p-*value^§^	%	*p-*value^§^
Males											
≥20 teeth	889	26.0 (23.0, 28.0)	< 0.001	546.1 (358.3, 757.2)	< 0.001	94.5	< 0.001	18.1	< 0.001	19.5	< 0.001
10–19 teeth	303	15.0(12.0, 17.0)		248.1 (139.3, 359.0)		66.0		22.1		23.1	
< 10 teeth	399	2.0(0,6.0)		115.0 (54.3, 199.5)		57.4		28.8		31.3	
≥20 teeth											
Eichner class A	783	26.0 (24.0, 28.0)	< 0.001	564.0 (369.8, 770.2)	< 0.001	94.4	0.824	18.1	1.000	19.8	0.601
Eichner class B/C	106	23.0 (22.0, 25.0)		442.9 (302.5, 618.8)		95.3		17.9		17.0	
10 to 19 teeth											
Eichner class A	229	14.0 (12.0, 17.0)	0.027	255.4 (155.0, 359.9)	0.475	68.1	0.204	19.2	0.037	24.5	0.428
Eichner class B/C	74	15.0 (13.0, 18.0)		224.8 (125.0, 357.3)		59.5		31.1		18.9	
< 10 teeth											
Eichner class A	377	1.0 (0, 5.0)	< 0.001	115.5 (54.3, 200.1)	0.531	57.8	0.511	28.4	0.469	31.3	1.000
Eichner class B/C	22	6.5 (2.0, 8.0)		85.5 (35.0, 201.8)		50.0		36.4		31.8	
Females											
≥20 teeth	840	26.0 (23.0, 27.0)	< 0.001	430.2 (267.5, 628.9)	< 0.001	94.4	< 0.001	15.8	0.301	27.7	0.183
10–19 teeth	316	16.0 (12.0, 18.0)		184.6 (110.7, 313.0)		68.0		18.4		23.1	
< 10 teeth	387	2.0 (0, 7.0)		92.3 (42.2, 156.8)		53.5		19.1		28.9	
≥20 teeth											
Eichner class A	716	26.0 (24.0, 28.0)	< 0.001	453.6 (285.7, 653.1)	< 0.001	95.4	0.006	15.9	1.000	27.0	0.233
Eichner class B/C	124	23.0 (22.0, 24.0)		312.6 (190.7, 467.8)		88.7		15.3		32.3	
10 to 19 teeth											
Eichner class A	250	15.0 (12.0, 18.0)	0.069	196.7 (122.8, 313.3)	0.026	68.8	0.656	19.2	0.582	24.4	0.328
Eichner class B/C	66	16.0 (13.8, 18.0)		142.9 (79.2, 313.3)		65.2		15.2		18.2	
< 10 teeth											
Eichner class A	379	2.0 (0, 7.0)	0.284	92.3 (42.3, 158.6)	0.457	53.6	1.000	19.5	0.362	29.6	0.111
Eichner class B/C	8	6.0 (0.25, 8.0)		81.8 (10.2, 118.0)		50.0		0		0	

Numerals indicate the median (lower quartile, upper quartile), or proportion

*BMI* Body mass index

^*^For continuous variables, differences between two groups were tested using the Mann-Whitney test, and those between three groups were tested using the Kruskal-Wallis test

^§^χ^2^ test used

As shown in Table 2, a significant difference in the proportion of individuals with serum albumin levels below 4.4 g/dL was found between Eichner rank A and ranks B/C (*p* = 0.037) only in males with 10 to 19 teeth, whereas in males with 20 or more teeth or fewer than 10 teeth, no significant difference was found in the proportion of individuals with serum levels below 4.4 g/dL or a BMI below 21.0 kg/m^2^. In females, no significant differences in the proportion of subjects with serum albumin levels below 4.4 g/dL or a BMI below 21.0 kg/m^2^ were found between Eichner rank A and ranks B/C.

As shown in Table 3, in males, the proportion of subjects with serum albumin levels below 4.4 g/dL increased significantly with decreasing maximum occlusal force, being 16.2, 19.1, 26.1, and 28.2% (*p* for trend < 0.001), and the proportion of subjects with a BMI below 21.0 kg/m^2^ also increased significantly, being 18.6, 21.4, 24.1, and 33.2% (*p* for trend < 0.001). The proportion of subjects with serum albumin levels below 4.4 g/dL and a BMI below 21.0 kg/m^2^ were significantly higher in the group in which all five food groups were non-masticable. In females, significant correlations were found between low maximum occlusal force and low serum albumin levels, and between all five food groups being non-masticable and a low BMI.

**Table 3:** Proportions with serum albumin below 4.4 g/dL and BMI below 21.0 kg/m^2^, classified by masticatory ability

		Serum albumin < 4.4 g/dL				BMI < 21.0 kg/m^2^			
	Number	Number	%	*p-*value^*^	*p-*for trend^§^	Number	%	*p-*value^*^	*p-*for trend^§^
Males (*n* = 1591)									
Occlusal force: ≥500 N	542	88	16.2	< 0.001	< 0.001	101	18.6	< 0.001	< 0.001
300–500 N	341	65	19.1			73	21.4		
100–300 N	449	117	26.1			108	24.1		
< 100 N	259	73	28.2			86	33.2		
All five food groups were masticable	1269	260	20.5	0.041		261	20.6	< 0.001	
All five food groups were non-masticable	322	83	25.8			107	33.2		
Females (*n* = 1543)									
Occlusal force: ≥500 N	357	44	12.3	0.016	0.002	87	24.4	0.595	0.200
300–500 N	338	57	16.9			92	27.2		
100–300 N	526	95	18.1			147	27.9		
< 100 N	322	69	21.4			92	28.6		
All five food groups were masticable	1215	207	17.0	0.805		311	25.6	< 0.014	
All five food groups were non-masticable	328	58	17.7			107	32.6		

*BMI* Body mass index

^*^χ^2^ test used

^§^The Mantel-extension test was used for the trend test

Tables 4 and 5 show the results of logistic regression analysis. Occlusal contact area showed a close correlation with maximum occlusal force (Spearman’s correlation coefficient: 0.973) and was, therefore, not entered as an independent variable. In males, no significant correlations were found among the number of teeth and masticatory ability with nutritional indices after adjustments for age, education length, alcohol consumption, smoking status, grip strength, and disease history were made (Table 4). Cancer (OR: 1.69; 95% CI: 1.18 to 2.43) showed a significant relationship with low BMI. Grip strength, hypertension, and dyslipidemia showed inverse relationships with low serum albumin levels and low BMIs. In females (Table 5), maximum occlusal force of 100 to 300 N (OR: 1.65; 95% CI: 1.06 to 2.55), a maximum occlusal force below 100 N (OR: 1.95; 95% CI: 1.15 to 3.31), and being an ex-smoker status (OR: 2.53; 95% CI: 1.42 to 4.50) showed significant correlations with serum albumin levels below 4.4 g/dL. Dyslipidemia showed an inverse relationship with a low albumin level. The masticability of foods in all five hardness groups, grip strength, hypertension, dyslipidemia, and diabetes showed inverse relationships with a low BMI.

**Table 4:** Results of logistic regression analysis with BMI and serum albumin as dependent variables among males *n* = 1591

	Serum albumin < 4.4 g/dL		BMI < 21.0 kg/m^2^	
Variables	Multivariate-adjusted odds ratio (95% CI)	*p*-value	Multivariate-adjusted odds ratio (95% CI)	*p*-value
Age, per 1-year increase	1.07 (1.04–1.10)	< 0.001	1.01 (0.99–1.04)	1.012
Education ≤ junior high school	0.95 (0.71–1.28)	0.750	0.78 (0.58–1.06)	1.113
Number of teeth (reference: ≥20)				
10–19	0.94 (0.64–1.37)	0.730	1.04 (0.70–1.55)	0.834
0–9	1.12 (0.75–1.67)	0.596	1.22 (0.80–1.85)	0.361
Functional teeth Eichner class B/C (reference: A)	1.38 (0.95–1.99)	0.089	0.81 (0.54–1.21)	0.300
Maximum occlusal force (reference: 500 N or more)				
300–500 N	1.09 (0.75–1.58)	0.656	1.13 (0.78–1.64)	0.527
100–300 N	1.38 (0.94–2.03)	0.103	0.94 (0.63–1.40)	0.745
< 100 N	1.17 (0.73–1.89)	0.518	0.93 (0.57–1.51)	0.774
All five food groups masticable (reference: all groups non-masticable)	1.09 (0.78–1.51)	0.619	0.73 (0.52–1.02)	0.065
Alcohol consumption (reference: no consumption)	1.19 (0.91–1.56)	0.194	0.73 (0.56–0.95)	0.020
Smoking status (reference: non-smoker)				
Ex-smoker	1.35 (0.97–1.88)	0.075	0.96 (0.69–1.31)	0.810
Smoker	1.22 (0.80–1.86)	0.349	1.48 (0.99–2.21)	0.058
Grip strength, per 10 kg increase	0.73 (0.58–0.91)	0.006	0.40 (0.31–0.51)	< 0.001
Hypertension	0.57 (0.44–0.75)	< 0.001	0.54 (0.41–0.71)	< 0.001
Dyslipidemia	0.71 (0.55–0.91)	0.008	0.33 (0.25–0.43)	< 0.001
Diabetes	1.16 (0.85–1.57)	0.349	0.56 (0.41–0.81)	0.002
Cancer	1.31 (0.92–1.89)	0.140	1.69 (1.18–2.43)	0.004
Myocardial infarction	0.91 (0.46–1.82)	0.797	0.39 (1.15–0.98)	0.045
Cerebral infarction	1.14 (0.71–1.83)	0.587	0.82 (0.49–1.39)	0.461
eGFR< 60	1.08 (0.81–1.44)	0.591	0.74 (0.54–1.00)	0.051

*BMI* Body mass index, *eGFR* estimated glomerular filtration rate

**Table 5:** Results of logistic regression analysis with BMI and serum albumin as dependent variables among females *n* = 1543

	Serum albumin < 4.4 g/dL		BMI < 21.0 kg/m^2^	
Variables	Multivariate-adjusted odds ratio (95% CI)	*p*-value	Multivariate-adjusted odds ratio (95% CI)	*p*- value
Age, per 1-year increase	1.05 (1.02–1.08)	< 0.003	1.00 (0.98–1.03)	1.003
Education ≤ junior high school	0.85 (0.63–1.15)	0.296	0.78 (0.60–1.01)	0.060
Number of teeth (reference: ≥20)				
10–19	0.97 (0.66–1.43)	0.885	0.68 (0.48–0.96)	0.030
0–9	0.79 (0.51–1.23)	0.293	0.80 (0.54–1.18)	0.260
Functional teeth Eichner class B/C (reference: A)	0.73 (0.47–1.14)	0.169	0.99 (0.68–1.42)	0.936
Maximum occlusal force (reference: 500 N or more)				
300–500 N	1.50 (0.97–2.33)	0.072	1.23 (0.86–1.76)	0.262
100–300 N	1.65 (1.06–2.55)	0.026	1.22 (0.85–1.76)	0.276
< 100 N	1.95 (1.15–3.31)	0.014	1.02 (0.65–1.62)	0.922
All five food groups masticable (reference: all groups non-masticable)	1.15 (0.80–1.66)	0.452	0.62 (0.46–0.85)	0.003
Alcohol consumption (reference: no consumption)	1.11 (0.76–1.61)	0.595	1.21 (0.88–1.67)	0.251
Smoking status (reference: non-smoker)				
Ex-smoker	2.53 (1.42–4.50)	0.002	0.86 (0.47–1.60)	0.640
Smoker	0.88 (0.36–2.15)	0.771	1.69 (0.85–3.36)	0.136
Grip strength, per 10 kg increase	0.90 (0.63–1.28)	0.566	0.41 (0.30–0.56)	< 0.001
Hypertension	0.77 (0.58–1.04)	0.086	0.67 (0.52–0.86)	0.002
Dyslipidemia	0.74 (0.56–0.98)	0.034	0.56 (0.44–0.71)	< 0.001
Diabetes	0.99 (0.64–1.52)	0.968	0.66 (0.44–0.99)	0.045
Cancer	0.91 (0.53–1.57)	0.745	0.95 (0.59–1.51)	0.812
Myocardial infarction	0.94 (0.31–2.85)	0.915	0.52 (0.17–1.60)	0.252
Cerebral infarction	1.52 (0.87–2.65)	0.144	0.58 (0.32–1.06)	0.077
eGFR< 60	1.12 (0.81–1.55)	0.502	0.96 (0.72–1.29)	0.793

*BMI* Body mass index, *eGFR* estimated glomerular filtration rate

## Discussion

In this study, the maximum occlusal force of 3134 community-dwelling people was measured using the Dental Prescale System. From Spearman’s correlation and the level of intra-class correlation, test-retest reliability was shown to be high. The large sample size and objective and subjective masticatory measurements make it is possible to generalize from the results of this study. In both males and females, the maximum occlusal force decreased significantly with the number of teeth, and the proportion of subjects for whom foods of all five hardness groups were masticable also decreased significantly.

In males, the proportion of subjects with serum albumin levels below 4.4 g/dL and with a BMI below 21.0 kg/m^2^ were significantly higher in the group with low objective masticatory ability and the group with low subjective masticatory ability. In females, significant correlations were found between low objective masticatory ability and low serum albumin levels, and between low subjective masticatory ability and a low BMI. In a previous study with adult subjects, a low number of teeth was reported to have a significant correlation with metabolic syndrome in females [28]. On the other hand, in a study with elderly people, a low number of teeth was found to be significantly associated with low body weight [29]. The findings in the present study were consistent with the latter research results. The theory that elderly people with a small number of teeth tend to have a lower objective and subjective masticatory ability, serum albumin levels, and BMI appears to be valid. A low BMI predicts the progression of mild cognitive impairment to dementia [30–33]. A BMI of < 23 is a risk factor for incidental functional disability due to dementia [34]. Maintaining masticatory ability and body weight may help prevent dementia. After adjusting for confounding factors in multivariate analysis, there was a significant association between masticatory ability and nutritional indicators in females. However, in males, a significant association was not observed between masticatory ability and nutritional indices. In men, it is thought that grip strength and internal diseases are largely involved in nutritional indicators.

It was expected that, among subjects with similar numbers of teeth, masticatory ability, serum albumin levels, and BMI would be lower in those in Eichner ranks B/C. However, in the present study, it was found that there was little difference in masticatory ability with and without occlusal support in the molar region, and no differences were found in the proportion of subjects with serum albumin levels of < 4.4 g/dL and a BMI of < 21.0 kg/m^2^. There was a limited recovery of masticatory ability after fitting dental prostheses.

Hypertension and dyslipidemia showed an inverse relationship with serum albumin levels below 4.4 g/dL and a BMI below 21.0 kg/m^2^. This finding supports the idea that being overweight or obese is a risk factor for hypertension and dyslipidemia. Smoker and ex-smoker status and a history of cancer showed significant correlations with low serum albumin levels and a low BMI. Grip strength showed an inverse relationship with low serum albumin levels and a low BMI. Not smoking, maintaining grip strength, and preventing cancer are also essential for preventing undernutrition.

In the present study, the numbers of dental implants were determined as artificial teeth. In contrast with dentures, which are not supported by the alveolar bone, dental implants are supported by the alveolar bone as a result of osseointegration, enabling them to generate high occlusal force. In the present study, 43 subjects (1.4%) had dental implants. In these 43 subjects, the lower quartile, median, and upper quartile of the number of implants were 2.0, 4.0, and 6.0. As the proportion of subjects with dental implants was so low, there is no need to consider the effect on the evaluation of subjects with dental implants having relatively high maximum occlusal forces despite having a small number of teeth.

Three limitations of our study merit consideration. The principal limitation was that the data were derived from a cross-sectional study; thus, we can only hypothesize for the biological credibility of the effect of tooth loss on undernutrition. Second, we did not consider reports from 24-h dietary recall, which could have presented the rationale for the relationship between the number of teeth and low undernutrition more precisely. Third, because of cost limitations, we did not investigate markers of inflammation in the gingival crevicular fluid or measure the alveolar bone loss, which could have helped in the evaluation of the severity of periodontal disease.

## Conclusion

A low number of teeth has a significant correlation with low masticatory ability, which has a significant correlation with low plasma albumin level and a low BMI. Not smoking, maintaining grip strength, preventing cancer, and masticatory ability are important for preventing undernutrition.

## Abbreviations

BMI: Body mass index
CI: Confidence interval
OR: Odds ratio
